# Crystal structure of bis­(1,3-di­amino­propane-κ^2^ *N*,*N*′)bis­[2-(4-nitro­phen­yl)acetato-κ*O*]cadmium

**DOI:** 10.1107/S2056989016000943

**Published:** 2016-01-23

**Authors:** Ian M. Rahn, Carlos L. Crawford, Zerihun Assefa, Jeffery Hendrich, Richard E. Sykora

**Affiliations:** a University of North Carolina–Chapel Hill, USA; b 1601 E Market St., Department of Chemistry, North Carolina A&T State University, Greensboro, NC 27411, USA; c University of South Alabama, Department of Chemistry, Mobile, AL 36688-0002, USA

**Keywords:** crystal structure, cadmium complex, 1,3-di­amino­propane, nitro­phenyl­acetic acid

## Abstract

In the structure of the title compound, [Cd(C_8_H_6_NO_4_)_2_(C_3_H_10_N_2_)_2_], the Cd^II^ atom is located on a center of symmetry with one independent Cd—O distance of 2.3547 (17) Å and two Cd—N distances of 2.3265 (18) and 2.3449 (19) Å. The Cd^II^ atom has an overall octa­hedral coordination environment. Several types of hydrogen-bonding inter­actions are evident. Both intra- and inter­molecular inter­actions occur between the amino groups and the O atoms of the acetate group. These N—H⋯O hydrogen bonds lead to a layered structure extending parallel to the *bc* plane. In addition, weak inter­molecular C—H⋯O inter­actions involving the nitro groups exist, leading to the formation of a three-dimensional network structure.

## Abstract 2

In a cadmium complex incorporating 1,3-di­amino­propane and nitro­phenyl­acetate ligands, the Cd^II^ atom is located on a center of symmetry with an overall octa­hedral coordination environment. Both intra- and inter­molecular inter­actions occur between the amino and acetate groups, leading to a layered structure.

## Chemical context   

The motivation for this study is based on the desire to expand the crystal engineering aspect of 1,3-di­amino propane and carboxyl­ate ligands and enhance their applications in host–guest chemistry (Sundberg *et al.*, 2001 ▸). It is known that the 1,3-di­amino­propane ligand behaves as a strong chelator and forms a stable six-membered ring in its metal complexes as well as being a good hydrogen-bond donor due to the existence of the amino groups (Sundberg *et al.*, 2001 ▸). In contrast, the 2-(4-nitro­phen­yl)acetate ligand has the potential to act as a linker and can also act as a good hydrogen-bond acceptor due to the four oxygen atoms it contains. Combination of these ligands in a single system has the potential to construct hydrogen-bond-directed supra­molecular networks. Herein, we report the synthesis and structure of the title compound, [Cd(C_8_H_6_NO_4_)_2_(C_3_H_10_N_2_)_2_], which displays such a hydrogen-bond-directed structure.
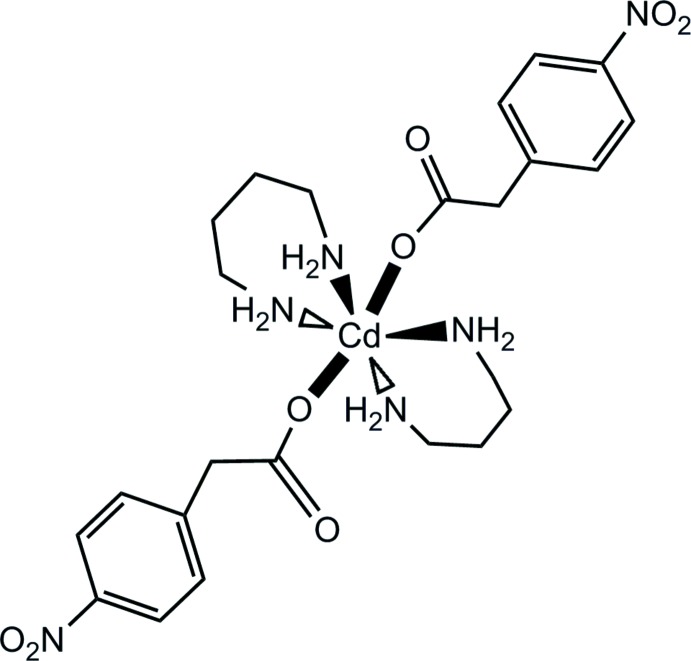


## Structural commentary   

**Figure 1:**
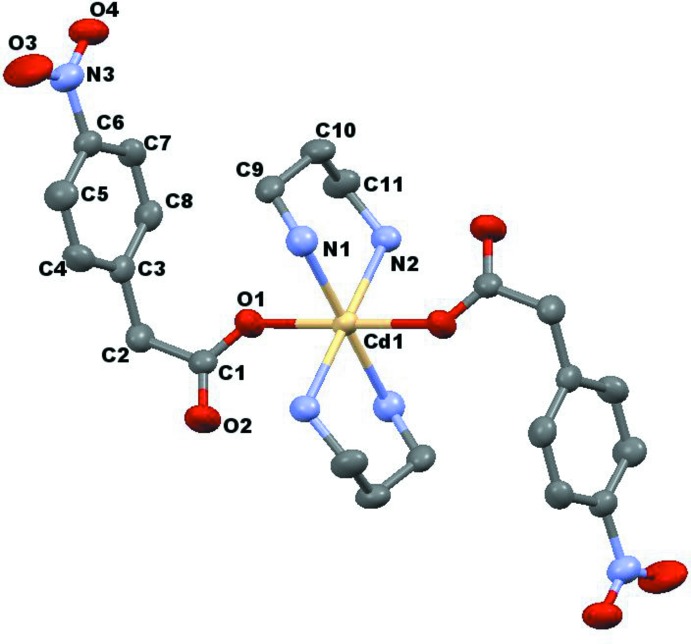
The mol­ecular structure of the title compound, with displacement ellipsoids drawn at the 50% probability level. Non-labelled atoms are generated by the symmetry code −*x* + 1, −*y* + 1, −*z* + 2.

As shown in Fig. 1 ▸, the Cd^II^ atom is located on a center of symmetry. Therefore the asymmetric unit consist of half of the mol­ecule. The Cd^II^ atom is octa­hedrally coordinated by four N atoms from two di­amino propane ligands and two O atoms of monodentate acetate groups from two nitro­phenyl-acetate ligands. The di­amino propane ligand shows a chelating coordination behavior and displays a chair conformation in the equatorial direction. This kind of coordination mode was also found in other similar complexes (Roberts *et al.*, 2015 ▸; Sundberg & Uggla, 1997 ▸ Sundberg *et al.*, 2001 ▸;), although the ligand has also been used as a linker of two metal atoms (Sheng *et al.*, 2014 ▸). The nitro group is slightly twisted out of the aromatic plane, with a dihedral angle of 3.6 (3)° between the two least-squares planes. A weak intra­molecular hydrogen bond of the type N—H⋯O involving one of the amino N atoms of the di­amino­propane ligand and the non-coordinating carboxyl­ate O atom of the nitro­phenyl­acetate ligand is evident in the structure at a distance of 3.029 (3) Å (Table 1 ▸).

## Supra­molecular features   

**Figure 2:**
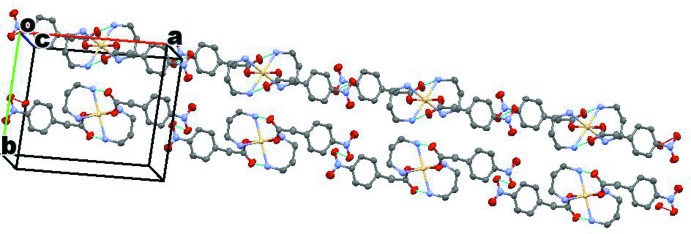
A packing diagram of the title compound. The light-blue dotted lines indicate intra­molecular hydrogen-bonding inter­actions, as well as intra­layer inter­actions involving the nitro groups of adjacent mol­ecules. A weak N—H⋯O inter­layer inter­action also exists at 3.149 (3) Å, linking the layers (see Table 1 ▸ for details).

Somewhat weaker inter­molecular N—H⋯O inter­actions involving the same types of donor and acceptor groups occur between neighboring mol­ecules (Table 1 ▸) and lead to a layered arrangement of the mol­ecules parallel to the *bc* plane (Fig. 2 ▸). It should be noted that one of the hydrogen atoms (H1*B*) of the amino group N1 has no acceptor group in its vicinity; the shortest donor⋯acceptor distance of N1—H1*B*⋯O2 = 3.868 Å seems to be too long for a significant inter­action. Several other weak inter­molecular hydrogen-bonding inter­actions of the C—H⋯O type also exist in the structure involving the O atoms of nitro groups and neighboring C—H groups.

## Synthesis and crystallization   

0.2 mmol (36.7 mg) of anhydrous CdCl_2_, 0.4 mmol (29.7 mg) of 1,3-di­amino­propane, and 0.4 mmol (72.5 mg) of 4-nitro­phenyl­acetic acid were added to 2 ml of methanol in a 5 ml beaker. The sample was covered with aluminum foil containing several small vent holes and left for a week to evaporate. The slow evaporation method was used to crystallize a colorless mononuclear species and crystals were gathered for X-ray crystallographic analysis.

## Refinement   

Crystal data, data collection and structure refinement details are summarized in Table 2 ▸. H atoms were placed in calculated positions and allowed to ride during subsequent refinement, with *U*_iso_(H) = 1.2*U*_eq_(C) and C—H distances of 0.93 Å for aromatic hydrogen atoms, *U*_iso_(H) = 1.2*U*_eq_(C) and C—H distances of 0.97 Å for methylene hydrogen atoms, and *U*_iso_(H) = 1.2*U*_eq_(N) and N—H distances of 0.90 Å for amino hydrogen atoms.

## Supplementary Material

Crystal structure: contains datablock(s) I. DOI: 10.1107/S2056989016000943/wm5258sup1.cif

Structure factors: contains datablock(s) I. DOI: 10.1107/S2056989016000943/wm5258Isup2.hkl

Supporting information file. DOI: 10.1107/S2056989016000943/wm5258Isup3.cdx

CCDC reference: 1447705

Additional supporting information:  crystallographic information; 3D view; checkCIF report

## Figures and Tables

**Table 1:** Hydrogen-bond geometry (Å, °)

*D*—H⋯*A*	*D*—H	H⋯*A*	*D*⋯*A*	*D*—H⋯*A*
N2—H2*A*⋯O2	0.90	2.23	3.029 (3)	147
N2—H2*B*⋯O2^i^	0.90	2.34	3.173 (3)	155
N1—H1*A*⋯O2^ii^	0.90	2.29	3.149 (3)	160
C5—H5⋯O4^iii^	0.93	2.50	3.253 (3)	139
C7—H7⋯O3^iv^	0.93	2.57	3.346 (3)	141
C10—H10*B*⋯O3^v^	0.97	2.69	3.629 (3)	163

Symmetry codes: (i) 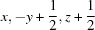; (ii) 
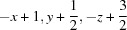
; (iii) 
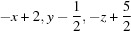
; (iv) 
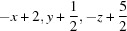
; (v) 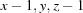.

**Table 2:** Experimental details

Crystal data	
Chemical formula	[Cd(C_8_H_6_NO_4_)_2_(C_3_H_10_N_2_)_2_]
*M*_r_	620.94
Crystal system, space group	Monoclinic, *P*2_1_/*c*
Temperature (K)	293
*a*, *b*, *c* (Å)	14.6943 (5), 11.1227 (3), 8.3523 (3)
β (°)	105.778 (4)
*V* (Å^3^)	1313.67 (7)
*Z*	2
Radiation type	Mo *K*α
μ (mm^−1^)	0.89
Crystal size (mm)	0.44 × 0.41 × 0.10

Data collection	
Diffractometer	Agilent Xcalibur Eos
Absorption correction	Multi-scan (*CrysAlis PRO*; Agilent, 2013 ▸)
*T*_min_, *T*_max_	0.923, 1.000
No. of measured, independent and observed [*I* > 2σ(*I*)] reflections	9750, 2400, 1911
*R*_int_	0.027
(sin θ/λ)_max_ (Å^−1^)	0.602

Refinement	
*R*[*F* ^2^ > 2σ(*F* ^2^)], *wR*(*F* ^2^), *S*	0.025, 0.056, 1.06
No. of reflections	2400
No. of parameters	170
H-atom treatment	H-atom parameters constrained
Δρ_max_, Δρ_min_ (e Å^−3^)	0.27, −0.26

Computer programs: *CrysAlis PRO* (Agilent, 2013 ▸), *OLEX2.solve* (Bourhis *et al.*, 2015 ▸), *SHELXL97* (Sheldrick, 2008 ▸)and *OLEX2* (Dolomanov *et al.*, 2009 ▸).

## supplementary crystallographic information

### Crystal data

**Table:**

[Cd(C_8_H_6_NO_4_)_2_(C_3_H_10_N_2_)_2_]	*F*(000) = 636
*M**_r_* = 620.94	*D*_x_ = 1.570 Mg m^−^^3^
Monoclinic, *P*2_1_/*c*	Mo *K*α radiation, λ = 0.71073 Å
*a* = 14.6943 (5) Å	Cell parameters from 3294 reflections
*b* = 11.1227 (3) Å	θ = 2.3–27.1°
*c* = 8.3523 (3) Å	µ = 0.89 mm^−^^1^
β = 105.778 (4)°	*T* = 293 K
*V* = 1313.67 (7) Å^3^	Plate, colourless
*Z* = 2	0.44 × 0.41 × 0.10 mm

### Data collection

**Table:**

Agilent Xcalibur Eos diffractometer	2400 independent reflections
Radiation source: Enhance (Mo) X-ray Source	1911 reflections with *I* > 2σ(*I*)
Graphite monochromator	*R*_int_ = 0.027
Detector resolution: 16.0514 pixels mm^-1^	θ_max_ = 25.4°, θ_min_ = 2.3°
ω scans	*h* = −17→17
Absorption correction: multi-scan (*CrysAlis PRO*; Agilent, 2013)	*k* = −12→13
*T*_min_ = 0.923, *T*_max_ = 1.000	*l* = −10→10
9750 measured reflections	

### Refinement

**Table:**

Refinement on *F*^2^	Secondary atom site location: difference Fourier map
Least-squares matrix: full	Hydrogen site location: inferred from neighbouring sites
*R*[*F*^2^ > 2σ(*F*^2^)] = 0.025	H-atom parameters constrained
*wR*(*F*^2^) = 0.056	*w* = 1/[σ^2^(*F*_o_^2^) + (0.0178*P*)^2^ + 0.5991*P*] where *P* = (*F*_o_^2^ + 2*F*_c_^2^)/3
*S* = 1.06	(Δ/σ)_max_ < 0.001
2400 reflections	Δρ_max_ = 0.27 e Å^−^^3^
170 parameters	Δρ_min_ = −0.26 e Å^−^^3^
0 restraints	Extinction correction: *SHELXL97* (Sheldrick, 2008), Fc^*^=kFc[1+0.001xFc^2^λ^3^/sin(2θ)]^-1/4^
Primary atom site location: iterative	Extinction coefficient: 0.0012 (2)

### Special details

**Table:**

Geometry. All e.s.d.'s (except the e.s.d. in the dihedral angle between two l.s. planes) are estimated using the full covariance matrix. The cell e.s.d.'s are taken into account individually in the estimation of e.s.d.'s in distances, angles and torsion angles; correlations between e.s.d.'s in cell parameters are only used when they are defined by crystal symmetry. An approximate (isotropic) treatment of cell e.s.d.'s is used for estimating e.s.d.'s involving l.s. planes.
Refinement. Refinement of *F*^2^ against ALL reflections. The weighted *R*-factor *wR* and goodness of fit *S* are based on *F*^2^, conventional *R*-factors *R* are based on *F*, with *F* set to zero for negative *F*^2^. The threshold expression of *F*^2^ > 2σ(*F*^2^) is used only for calculating *R*-factors(gt) *etc*. and is not relevant to the choice of reflections for refinement. *R*-factors based on *F*^2^ are statistically about twice as large as those based on *F*, and *R*- factors based on ALL data will be even larger.

### Fractional atomic coordinates and isotropic or equivalent isotropic displacement parameters (Å^2^)

**Table:**

	*x*	*y*	*z*	*U*_iso_*/*U*_eq_	
Cd1	0.5000	0.5000	1.0000	0.03617 (10)	
O1	0.62078 (12)	0.49421 (16)	0.8639 (2)	0.0497 (5)	
C9	0.30006 (17)	0.5182 (2)	0.7208 (3)	0.0405 (6)	
H9A	0.2571	0.5602	0.6291	0.049*	
H9B	0.2751	0.5245	0.8167	0.049*	
O2	0.57547 (14)	0.35381 (19)	0.6686 (2)	0.0639 (6)	
C1	0.63446 (17)	0.4232 (2)	0.7568 (3)	0.0359 (6)	
N2	0.44583 (14)	0.31282 (16)	0.8902 (2)	0.0387 (5)	
H2A	0.4749	0.2947	0.8113	0.046*	
H2B	0.4642	0.2579	0.9717	0.046*	
C8	0.84336 (18)	0.5357 (2)	0.9702 (3)	0.0393 (6)	
H8	0.8149	0.6073	0.9248	0.047*	
C3	0.81242 (16)	0.4281 (2)	0.8897 (3)	0.0339 (5)	
C7	0.91540 (18)	0.5390 (2)	1.1161 (3)	0.0386 (6)	
H7	0.9355	0.6116	1.1690	0.046*	
C6	0.95687 (16)	0.4323 (2)	1.1816 (3)	0.0354 (6)	
C4	0.85551 (18)	0.3230 (2)	0.9607 (3)	0.0432 (6)	
H4	0.8355	0.2500	0.9089	0.052*	
C2	0.73382 (17)	0.4241 (3)	0.7300 (3)	0.0426 (6)	
H2C	0.7414	0.3527	0.6682	0.051*	
H2D	0.7394	0.4935	0.6628	0.051*	
N3	1.03724 (15)	0.4348 (2)	1.3320 (3)	0.0465 (6)	
C5	0.92748 (19)	0.3240 (2)	1.1067 (3)	0.0457 (7)	
H5	0.9555	0.2526	1.1533	0.055*	
C11	0.34305 (18)	0.2995 (2)	0.8167 (3)	0.0502 (7)	
H11A	0.3109	0.3124	0.9025	0.060*	
H11B	0.3297	0.2180	0.7757	0.060*	
C10	0.30436 (19)	0.3869 (2)	0.6752 (3)	0.0482 (7)	
H10A	0.3428	0.3806	0.5978	0.058*	
H10B	0.2409	0.3613	0.6167	0.058*	
O4	1.06568 (14)	0.53165 (17)	1.3946 (2)	0.0551 (5)	
O3	1.07278 (16)	0.33928 (19)	1.3882 (3)	0.0844 (8)	
N1	0.39369 (14)	0.57670 (19)	0.7594 (2)	0.0432 (5)	
H1A	0.3865	0.6561	0.7727	0.052*	
H1B	0.4183	0.5668	0.6727	0.052*	

### Atomic displacement parameters (Å^2^)

**Table:**

	*U*^11^	*U*^22^	*U*^33^	*U*^12^	*U*^13^	*U*^23^
Cd1	0.02686 (14)	0.03292 (15)	0.04385 (16)	−0.00022 (12)	0.00131 (10)	−0.00811 (12)
O1	0.0383 (10)	0.0604 (12)	0.0531 (11)	−0.0088 (9)	0.0174 (8)	−0.0232 (10)
C9	0.0345 (14)	0.0490 (16)	0.0338 (12)	0.0065 (12)	0.0017 (11)	0.0014 (11)
O2	0.0476 (12)	0.0746 (14)	0.0667 (13)	−0.0169 (11)	0.0109 (10)	−0.0331 (11)
C1	0.0340 (14)	0.0388 (14)	0.0310 (12)	0.0009 (12)	0.0024 (11)	−0.0012 (11)
N2	0.0410 (12)	0.0323 (11)	0.0375 (11)	0.0024 (10)	0.0017 (9)	−0.0008 (9)
C8	0.0380 (14)	0.0317 (13)	0.0472 (15)	0.0046 (11)	0.0097 (12)	0.0054 (11)
C3	0.0278 (12)	0.0433 (14)	0.0331 (12)	−0.0008 (12)	0.0126 (10)	0.0003 (11)
C7	0.0371 (14)	0.0296 (12)	0.0486 (15)	−0.0051 (11)	0.0107 (12)	−0.0057 (11)
C6	0.0300 (13)	0.0364 (14)	0.0388 (13)	−0.0041 (11)	0.0072 (11)	0.0001 (11)
C4	0.0467 (16)	0.0331 (14)	0.0469 (15)	−0.0065 (13)	0.0080 (13)	−0.0086 (12)
C2	0.0382 (15)	0.0568 (17)	0.0345 (13)	0.0017 (14)	0.0126 (11)	−0.0033 (12)
N3	0.0389 (13)	0.0500 (14)	0.0453 (13)	−0.0026 (12)	0.0023 (11)	0.0017 (11)
C5	0.0496 (17)	0.0301 (13)	0.0506 (16)	0.0023 (13)	0.0020 (13)	0.0055 (12)
C11	0.0450 (17)	0.0375 (14)	0.0589 (17)	−0.0096 (13)	−0.0015 (14)	−0.0011 (13)
C10	0.0440 (16)	0.0464 (16)	0.0428 (15)	−0.0008 (13)	−0.0077 (12)	−0.0060 (12)
O4	0.0533 (12)	0.0542 (12)	0.0502 (11)	−0.0163 (10)	0.0013 (9)	−0.0093 (9)
O3	0.0824 (17)	0.0538 (13)	0.0835 (16)	0.0104 (13)	−0.0346 (13)	0.0066 (12)
N1	0.0452 (13)	0.0394 (12)	0.0439 (12)	0.0007 (11)	0.0101 (10)	0.0025 (10)

### Geometric parameters (Å, º)

**Table:**

Cd1—O1^i^	2.3547 (17)	C3—C2	1.509 (3)
Cd1—O1	2.3547 (17)	C7—H7	0.9300
Cd1—N2^i^	2.3265 (18)	C7—C6	1.377 (3)
Cd1—N2	2.3265 (18)	C6—N3	1.472 (3)
Cd1—N1^i^	2.3449 (19)	C6—C5	1.373 (3)
Cd1—N1	2.3449 (19)	C4—H4	0.9300
O1—C1	1.250 (3)	C4—C5	1.379 (3)
C9—H9A	0.9700	C2—H2C	0.9700
C9—H9B	0.9700	C2—H2D	0.9700
C9—C10	1.515 (3)	N3—O4	1.220 (3)
C9—N1	1.476 (3)	N3—O3	1.220 (3)
O2—C1	1.242 (3)	C5—H5	0.9300
C1—C2	1.536 (3)	C11—H11A	0.9700
N2—H2A	0.9000	C11—H11B	0.9700
N2—H2B	0.9000	C11—C10	1.516 (3)
N2—C11	1.475 (3)	C10—H10A	0.9700
C8—H8	0.9300	C10—H10B	0.9700
C8—C3	1.387 (3)	N1—H1A	0.9000
C8—C7	1.380 (3)	N1—H1B	0.9000
C3—C4	1.384 (3)		
			
O1—Cd1—O1^i^	180.0	C6—C7—C8	118.6 (2)
N2—Cd1—O1	90.41 (7)	C6—C7—H7	120.7
N2—Cd1—O1^i^	89.59 (7)	C7—C6—N3	119.3 (2)
N2^i^—Cd1—O1^i^	90.41 (7)	C5—C6—C7	121.6 (2)
N2^i^—Cd1—O1	89.59 (7)	C5—C6—N3	119.0 (2)
N2—Cd1—N2^i^	180.0	C3—C4—H4	119.2
N2—Cd1—N1^i^	95.11 (7)	C5—C4—C3	121.6 (2)
N2^i^—Cd1—N1	95.11 (7)	C5—C4—H4	119.2
N2^i^—Cd1—N1^i^	84.89 (7)	C1—C2—H2C	108.8
N2—Cd1—N1	84.89 (7)	C1—C2—H2D	108.8
N1—Cd1—O1^i^	89.42 (7)	C3—C2—C1	113.60 (19)
N1^i^—Cd1—O1^i^	90.58 (7)	C3—C2—H2C	108.8
N1^i^—Cd1—O1	89.42 (7)	C3—C2—H2D	108.8
N1—Cd1—O1	90.58 (7)	H2C—C2—H2D	107.7
N1—Cd1—N1^i^	180.00 (7)	O4—N3—C6	119.0 (2)
C1—O1—Cd1	130.67 (16)	O4—N3—O3	122.9 (2)
H9A—C9—H9B	107.9	O3—N3—C6	118.2 (2)
C10—C9—H9A	109.1	C6—C5—C4	118.7 (2)
C10—C9—H9B	109.1	C6—C5—H5	120.7
N1—C9—H9A	109.1	C4—C5—H5	120.7
N1—C9—H9B	109.1	N2—C11—H11A	109.1
N1—C9—C10	112.3 (2)	N2—C11—H11B	109.1
O1—C1—C2	116.4 (2)	N2—C11—C10	112.6 (2)
O2—C1—O1	126.5 (2)	H11A—C11—H11B	107.8
O2—C1—C2	117.1 (2)	C10—C11—H11A	109.1
Cd1—N2—H2A	108.0	C10—C11—H11B	109.1
Cd1—N2—H2B	108.0	C9—C10—C11	117.0 (2)
H2A—N2—H2B	107.3	C9—C10—H10A	108.0
C11—N2—Cd1	117.10 (15)	C9—C10—H10B	108.0
C11—N2—H2A	108.0	C11—C10—H10A	108.0
C11—N2—H2B	108.0	C11—C10—H10B	108.0
C3—C8—H8	119.3	H10A—C10—H10B	107.3
C7—C8—H8	119.3	Cd1—N1—H1A	109.0
C7—C8—C3	121.5 (2)	Cd1—N1—H1B	109.0
C8—C3—C2	121.6 (2)	C9—N1—Cd1	113.02 (14)
C4—C3—C8	118.0 (2)	C9—N1—H1A	109.0
C4—C3—C2	120.4 (2)	C9—N1—H1B	109.0
C8—C7—H7	120.7	H1A—N1—H1B	107.8
			
Cd1—O1—C1—O2	17.7 (4)	C3—C8—C7—C6	0.1 (4)
Cd1—O1—C1—C2	−163.60 (16)	C3—C4—C5—C6	−0.4 (4)
Cd1—N2—C11—C10	58.8 (3)	C7—C8—C3—C4	0.5 (4)
O1^i^—Cd1—N2—C11	46.37 (18)	C7—C8—C3—C2	−179.9 (2)
O1—Cd1—N2—C11	−133.63 (18)	C7—C6—N3—O4	−0.5 (4)
O1—Cd1—N1—C9	135.70 (16)	C7—C6—N3—O3	179.8 (3)
O1^i^—Cd1—N1—C9	−44.30 (16)	C7—C6—C5—C4	1.0 (4)
O1—C1—C2—C3	43.5 (3)	C4—C3—C2—C1	95.2 (3)
O2—C1—C2—C3	−137.7 (2)	C2—C3—C4—C5	−180.0 (2)
N2^i^—Cd1—O1—C1	−170.6 (2)	N3—C6—C5—C4	−176.5 (2)
N2—Cd1—O1—C1	9.4 (2)	C5—C6—N3—O4	177.1 (2)
N2—Cd1—N1—C9	45.35 (16)	C5—C6—N3—O3	−2.6 (4)
N2^i^—Cd1—N1—C9	−134.65 (16)	C10—C9—N1—Cd1	−65.9 (2)
N2—C11—C10—C9	−71.0 (3)	N1^i^—Cd1—O1—C1	104.5 (2)
C8—C3—C4—C5	−0.3 (4)	N1—Cd1—O1—C1	−75.5 (2)
C8—C3—C2—C1	−84.5 (3)	N1^i^—Cd1—N2—C11	136.92 (18)
C8—C7—C6—N3	176.6 (2)	N1—Cd1—N2—C11	−43.08 (18)
C8—C7—C6—C5	−0.8 (4)	N1—C9—C10—C11	76.7 (3)

Symmetry code: (i) −*x*+1, −*y*+1, −*z*+2.

### Hydrogen-bond geometry (Å, º)

**Table:**

*D*—H···*A*	*D*—H	H···*A*	*D*···*A*	*D*—H···*A*
N2—H2*A*···O2	0.90	2.23	3.029 (3)	147
N2—H2*B*···O2^ii^	0.90	2.34	3.173 (3)	155
N1—H1*A*···O2^iii^	0.90	2.29	3.149 (3)	160
C5—H5···O4^iv^	0.93	2.50	3.253 (3)	139
C7—H7···O3^v^	0.93	2.57	3.346 (3)	141
C10—H10*B*···O3^vi^	0.97	2.69	3.629 (3)	163

Symmetry codes: (ii) *x*, −*y*+1/2, *z*+1/2; (iii) −*x*+1, *y*+1/2, −*z*+3/2; (iv) −*x*+2, *y*−1/2, −*z*+5/2; (v) −*x*+2, *y*+1/2, −*z*+5/2; (vi) *x*−1, *y*, *z*−1.
